# Probing the Structure, Cytocompatibility, and Antimicrobial Efficacy of Silver-, Strontium-, and Zinc-Doped Monetite

**DOI:** 10.1021/acsabm.2c00047

**Published:** 2022-03-24

**Authors:** Alaa Adawy, Raquel Diaz

**Affiliations:** † Unit of Electron Microscopy and Nanotechnology, Institute for Scientific and Technological Resources (SCTs), University of Oviedo, 33006 Oviedo, Asturias, Spain; ‡ Nanomaterials and Nanotechnology Research Centre—CINN (CSIC), 33940 El Entrego, Spain

**Keywords:** biocompatible materials, calcium phosphate, monetite, brushite, antimicrobial, silver, zinc, strontium

## Abstract

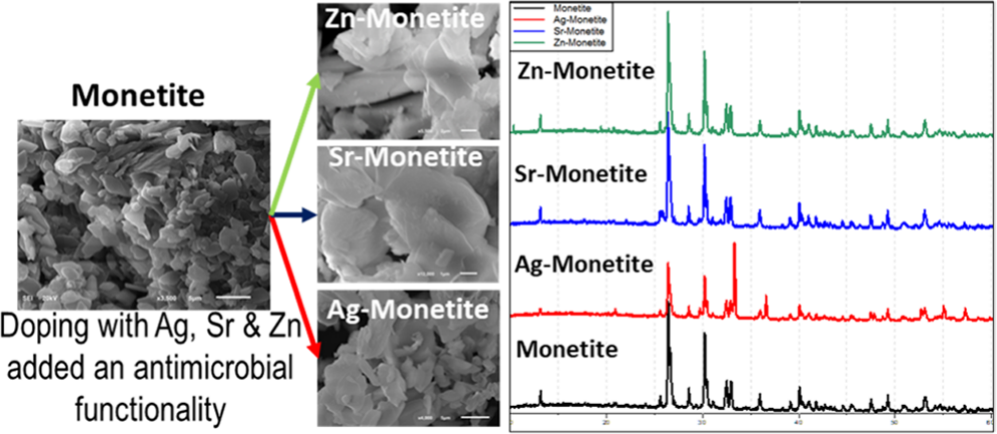

Calcium phosphate phases are among the most widely accepted compounds for biomaterial applications, of which the resorbable phases have gained particular attention in recent years. Brushite and its anhydrous form monetite are among the most interesting resorbable calcium phosphate phases that can be applied as cements and for *in situ* fabrication of three-dimensional (3D) implants. Of these two dicalcium phosphate compounds, monetite is more stable and undergoes slower degradation than brushite. The purpose of the current study is to synthesize and dope monetite with the antimicrobial elements silver and zinc and the osteoinductive element strontium and investigate the possible structural variations as well as their biocompatibility and antimicrobial effectiveness. For this, powder X-ray diffraction (PXRD), energy-dispersive X-ray spectroscopy (EDX), scanning electron microscopy (SEM), and cryo-transmission electron microscopy (cryo-TEM) were used to thoroughly study the synthesized structures. Moreover, the ASTM E-2149-01 protocol and a cell proliferation assay were used to determine the minimum inhibitory concentration (MIC) and minimum bactericidal concentration (MBC) and the cytocompatibility of the different phases with the Soas-2 cell line, respectively. The results confirm the successful synthesis and doping procedures, such that zinc was the most incorporated element into the monetite phase and strontium was the least incorporated element. The microbiological studies revealed that silver is a very effective antimicrobial agent at low concentrations but unsuitable at high concentrations because its cytotoxicity would prevail. On the other hand, doping the compounds with zinc led to a reasonable antimicrobial activity without compromising the biocompatibility to obviously high concentrations. The study also highlights that strontium, widely known for its osteoinductivity, bears an antimicrobial effect at high concentrations. The generated doped compounds could be beneficial for prospective studies as bone cements or for scaffold biomaterial applications.

## 1 Introduction

In recent decades, the role of biomaterials has advanced from being solely biocompatible materials that would replace biological tissue without triggering adverse effects (bioinert) to being bioactive functional materials that would also stimulate the body to regenerate its function (healing or therapeutic effect) or have precautionary effects (antimicrobial).^1−3^ This drastically increased the importance of biocompatible resorbable materials and the investigations on the safe usage of antimicrobial precautionary elements such as silver, zinc, iron, and gold.^4−9^

Among the most interesting materials in terms of their biocompatibility and similarity to hard tissue are metal phosphates, especially calcium phosphate compounds (CPCs),^10−12^ which can be applied as either bone cement^13,14^ or coating layers for bioinert implants.^15−21^ For instance, crystalline hydroxyapatite (HA) with the formula Ca_10_(PO_4_)_6_(OH)_2_ is the closest CPC to the biological apatite with the formula Ca_5_(PO_4_)_2.5_(CO_3_)_0.5_(OH), which could vary in size, perfection, and concentration of minor components (e.g., carbonate and magnesium) and constitutes the mineral phases of teeth and bones.^22−24^ In addition, some other forms of doped apatite, such as fluor-carbonate hydroxyapatite, are found in fish enameloids.^25^ Other biologically relevant CPCs are amorphous calcium phosphate (ACP),^26,27^ monetite (dibasic calcium phosphate anhydrate, DCPA; CaHPO_4_),^28,29^ brushite (dibasic calcium phosphate dihydrate, DCPD; CaHPO_4_.2H_2_O),^30^ octacalcium phosphate (OCP); Ca_8_H_2_(PO_4_)_6_.H_2_O),^31^ calcium pyrophosphate; Ca_2_P_2_O_7_),^32^ α-tricalcium phosphate (α-TCP),^33^ β-tricalcium phosphate (β-TCP; Ca_3_(PO_4_)_2_),^34^ and tetracalcium phosphate; Ca_4_(PO_4_)_2_O).^35^ Nevertheless, HA is not a resorbable form of CPC because the resorptive capacity of CPCs increases with their reduced crystallinity, such that ACP ≫ brushite > monetite > OCP > α-TCP ≫ β-TCP > HA.^36^ Resorbable CPCs are characterized by their ability to be broken down and absorbed by the body over time. The main challenge with resorbable CPCs is the interplay between the resorption rate and the growth of new bone tissues.^37^ Resorbable CPCs have several uses throughout the body in skeletal and dental reconstruction that fall into three major categories: guided bone regeneration, coatings or cements, and timed release of medicines.^38−40^ Among the most interesting resorbable CPCs that can be used as cements and for the fabrication of three-dimensional (3D) implants *in situ* are brushite and monetite.^41,42^ These two CPCs can resorb under physiological conditions at rates that still allow for successful body recovery.^43^ Despite their similar chemical composition, their *in vivo* interactions are quite different owing to differences in water solubility at a physiological pH.^44^ Interestingly, brushite can be easily converted through dehydration into monetite, which has the advantage of being a more stable phase that undergoes slower degradation when compared to brushite.^45^ Therefore, monetite is used as a component of hydroxyapatite cements for orthopedic applications because it is a better alternative than brushite in bone regeneration processes. Unlike brushite that tends to reprecipitate out as insoluble HA with limited bioresorpability, monetite does not reprecipitate into HA *in vivo*, and many studies have reported its good osteoconductivity and osteoinductivity.^46^ Moreover, adequately customized monetite biomaterials can be prepared in different shapes and sizes through thermal treatment of brushite preset cements.^47^

As mentioned earlier, it is nowadays a necessity to seek biocompatibility and bioactivity side by side with antimicrobial activity in biomaterials.^48,49^ Since we are already in the phase of increasing antibiotic resistance, researchers are seeking other effective elements that may provide another means for attacking microbes at the nanoscale. Among these elements are silver and zinc.

Silver is widely known for its effectiveness as an antimicrobial agent against Gram-positive and Gram-negative bacteria, fungi, protozoa, and certain viruses including antibiotic-resistant strains.^50^ Most of the research on silver antimicrobial capabilities deals with it as nanoparticles. However, recent studies implied that silver is more effective in its ionized form, Ag^+^, through which it causes damage to cells by interacting with thiol-containing proteins and DNA.^51,52^ On the other hand, zinc is the old-but-gold element in terms of its antimicrobial effects against various bacterial and fungal strains and its applications.^53^ Zinc is considered as nature’s antimicrobial agent through which our bodies attack any microbial infection.^54^ Although most studies have focused on the application of zinc oxide nanoparticles for antimicrobial activity, several investigations have highlighted the significant effect of Zn^2+^ in that context.^55,56^ Nonetheless, strontium, with its outstanding capabilities to induce osteoinductivity and to regulate the release of silver nanoparticles as was reported recently, has been scarcely investigated for its antimicrobial activity.^57−60^

Therefore, the purpose of this study is to synthesize monetite phases doped with the biofunctional elements silver, zinc, and strontium. In addition to the structural studies, an assessment of the antimicrobial activity and cytocompatibility of the resultant Ag-/Zn-/Sr-doped monetite and phosphate phases is reported. The results demonstrate the possibility of relying on strontium as an antimicrobial agent at relatively elevated concentrations. Moreover, the study shows that in addition to the successful doping of monetite phases, their potential antimicrobial activities did not greatly affect their cytocompatibilities, except in the case of silver, which, in turn, exhibited a very good antimicrobial effect at significantly low concentrations.

## 2 Materials and Methods

### 2.1 Preparation of Monetite and Their Doping with Silver, Strontium, and Zinc (M, Ag-M, Sr-M, Zn-M)

All of the used chemicals were purchased from Sigma-Aldrich and were used without further purification. The preparation procedure consisted of two stages: preparation of the calcium phosphate phase [a], followed by doping of the resultant monetite phase with silver, strontium, and zinc. For the monetite preparation, β-tricalcium phosphate [Ca_3_(PO_4_)_2_, purity ≥ 98%] was mixed with monocalcium phosphate monohydrate [Ca(H_2_PO_4_)_2_·H_2_O, purity ≥ 85%] at a ratio of 1.2:1 in a mortar with a powder to distilled water ratio of 3:1. The resultant paste was thoroughly mixed until complete homogeneity was achieved and left in an oven preheated to 100 °C and readjusted to 50 °C overnight.

For doping the resultant monetite powders with silver, strontium, and zinc, 20 mL of 0.25 M silver nitrate [AgNO_3_, purity ≥ 99%], strontium nitrate [Sr(NO_3_)_2_, purity ≥ 99%], and zinc nitrate hexahydrate [Zn(NO_3_)_2_.6H_2_O, purity ≥ 98%] prepared in distilled water were mixed (under stirring) with adequate amounts of the resultant monetite powder for 30 min and put in Teflon-lined stainless-steel hydrothermal autoclave reactors (40 mL) overnight at 100 °C. The molar ratio of the calcium to Ag/Zn/Sr was adjusted to 1:0.15. Afterward, the resultant different powders were centrifuged, washed with distilled water, filtrated from the supernatant solution, and dehydrated in an oven at 40 °C overnight (24 h). For the other phosphate phases, the Ag/Zn/Sr to calcium ratio was adjusted to 1:1; otherwise, the same synthesis procedure was followed.

### 2.2 Structural Characterization Methodologies

The powder X-ray diffraction (PXRD) patterns were recorded on an X’Pert diffractometer (Philips, Amelo, the Netherlands) with Cu Kα radiation (λ = 1.5406 Å) at room temperature over the angular 2θ range 5–75° with a step of 0.02° and a counting time of 0.4 s/step. Top-view scanning electron microscopy (SEM) micrographs for secondary and back-scattered electrons (SE and BSE) and energy-dispersive X-ray microanalysis (EDX) were recorded with a JEOL JSM-6100 scanning electron microscope (JEOL, Tokyo, Japan) operating at 20 kV coupled with an X-Max silicon drift detector (SDD) 80 mm^2^ energy-dispersive X-ray spectroscopy (EDS) detector (Oxford Instruments, High Wycombe, England). The high-resolution transmission electron microscopy (HRTEM) studies were performed under cryogenic conditions on a JEOL JEM-2100F transmission electron microscope (JEOL, Tokyo, Japan) operating at an accelerating voltage of 200 kV and equipped with a field-emission gun and an ultra-high-resolution pole-piece that provided a point resolution better than 1.9 Å. This TEM is also equipped with a scanning transmission electron microscope (STEM) control unit (Gatan), energy-dispersive X-ray spectroscopy (EDS) detector (Oxford Instruments, X-Max (SDD) 80 mm^2^, High Wycombe, England), CCD camera (Gatan 14-bit Orius SC600, GATAN, Pleasanton), and bright-field (BF) and high-angle annular dark-field (HAADF) detectors (JEOL). This microscope was used to perform TEM, HRTEM, selected area electron diffraction (SAED), STEM (BF and HAADF), and EDX-STEM (line-scan and area mapping) analysis. Fine powder of every sample was dispersed in ethanol, briefly sonicated, and sprayed on a Lacey-carbon-on-copper grid (200 mesh, EM science, Hatfield, England) and then allowed to air dry. Afterward, the dried grids were mounted on a JEOL cryo-holder. All micrographs were acquired, processed, and analyzed using the suite of Gatan Digital Micrograph software (version 2.32.888.0). Quantitative analyses were done using INCA Microanalysis software (version 4.15).

### 2.3 Investigating the Antimicrobial Activity of All Synthesized Phases

Two parameters were determined: minimum inhibitory concentration (MIC) and minimum bactericidal concentration (MBC). The MIC of a certain agent is the minimum concentration of the agent at which no microorganism development is observed. The MBC is the minimum concentration that can kill a bacterial strain (death of 99.9% of the inoculum). The protocol ASTM E-2149-01 (Antimicrobial Activity of Immobilized Agents Under Dynamic Contact Conditions) describes a test method that allows the measurement of the antimicrobial activity of a material or product under dynamic contact conditions. The purpose of this test is to examine the antimicrobial activity of a material against a suspension of a particular microorganism. This is achieved by exposing the microorganism to the material to be tested under dynamic conditions (agitation) and analyzing the number of surviving microorganisms after certain times. In this case, the microorganism is exposed to a series of serial dilutions of the agent. Before starting the experiment, the material to be tested was sterilized in an autoclave at 121 °C for 20 min. The strain used as inoculum was *Escherichia coli* ATCC 8739 at an initial concentration of 1.2 × 10^6^ CFU/mL (CFU: colony-forming unit). To determine the MIC, liquid nutrient broth (NB) was used as culture media in which concentrations of 25, 50, and 75 mg/mL of all phases (Ag-M, Ag-P, Sr-M, Sr-P, Zn-M, Zn-P) were introduced. The experiments were run in triplicate. The tubes were placed in an incubator overnight (≈24 h) at 37 °C. After the incubation period, the tubes that showed turbidity were discarded as this indicates bacterial growth. The clear tubes with the lowest product concentration indicated the MIC. The solutions in all clear tubes were then used to prepare serial dilutions (10^–1^ down to 10^–8^) of every concentration, and this time, the serial dilution was performed in Petri dishes containing solid NB (1.5% agar). Afterward, the prepared Petri dishes were incubated for another 24 h at 37 °C. A decrease in the number of counted colonies proportional to the dilution factor is a good indication of the success of the experiment. The Petri dishes with no observed microbial colonies (completely clear) indicate the concentration of the tested material corresponding to the MBC.

### 2.4 Studying Cell Proliferation in the Presence of All Synthesized Phases

The Saos-2 cell line (Sarcoma osteogenic),purchased from LGC (product code: ATCC-HTB-85), was used in this assay. The cells were cultured in a complete Dulbecco′s modified Eagle′s medium (DMEM) containing 10% fetal bovine serum (FBS) and 1% penicillin/streptomycin at 37 °C in a humidified incubator containing 5% CO_2_. A Cell Counting Kit 8 (CCK-8; Sigma, Missouri) was used to determine the cell viability of Saos-2. The cells were seeded at 1 × 10^3^/well into a 96-well plate and cultured for 24 h in a DMEM-10% FBS growth medium. The experiments were designed in triplicate to confirm their reproducibility. Equal masses of the phases’ powders were then placed in contact with the cells, and serial dilutions were prepared. The microplate was then incubated for 3–7 days. The powders were filtered out, and 100 μL of DMEM-10% FBS and 10 μL of CCK-8 reagent were added to each well and incubated for an additional 3 h. The absorbance of each well was determined at wavelength 450 nm in an Elx800 plate reader (Biotek, Winooski). The obtained absorbance value was proportional to the survival of the cells. The cell viability rate was determined by calculating the percentage ratio of the absorbance at wavelength 450 nm from a well of a given powder concentration to the absorbance from a control well with cells without powder.

## 3 Results

### 3.1 Structural Aspects

As will be shown below, the doping process at low molar ratios led to the formation of monetite doped with silver, strontium, and zinc ions, hereafter called Ag-monetite (Ag-M), Sr-monetite (Sr-M), and Zn-monetite (Zn-M). At 1:1 molar ratios, the hydrolysis of monetite took place at least after the treatment with AgNO_3_ and Zn(NO_3_)_2_.6H_2_O, leading to the formation of other phosphate phases containing the three ions; hereafter they will be indicated as Ag-phosphate (Ag-P), Sr-phosphate (Sr-P), and Zn-phosphate (Zn-P). The resultant metal phosphate samples were collected in the form of powders. Except for the silver-doped phases that have a light-yellowish color, Ag-P has a darker color compared to Ag-M; all other phases have whitish colors.

**Figure 1:**
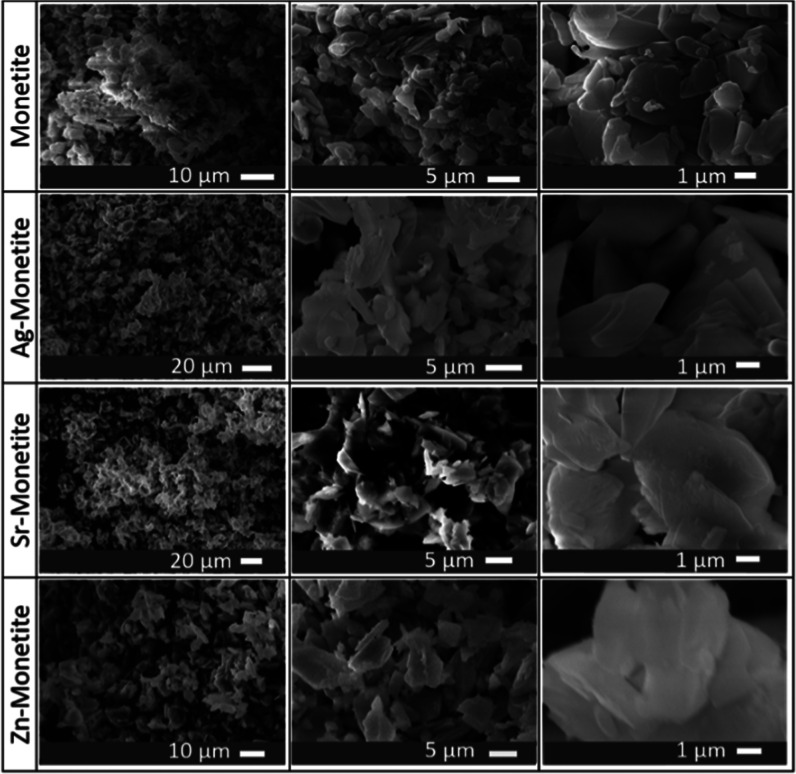
SEM micrographs at increasing magnifications (per column) for the monetite platelike microcrystals (1st row) and their changes after being doped with silver (2nd row) Ag-M, strontium (3rd row) Sr-M, and zinc (4th row) Zn-M.

The microscopic inspection using a scanning electron microscope (SEM) revealed that all monetite phases have the same morphology of stacked platelike microcrystalline structures with no observed morphological variations between the pristine phase and its doped counterparts (Figure 1). On the other hand, Ag-P bears different morphologies in the form of much smaller granules, proving that calcium has been completely substituted with Ag after complete hydrolysis of the monetite in a AgNO_3_ solution, whereas Sr-P and Zn-P possessed a morphology very similar to pristine monetite and its doped phases (Figure S1).

Energy-dispersive X-ray analysis combined with scanning transmission electron microscopy (STEM) and SEM, for high- and low-magnification analyses, respectively, showed that the calcium to phosphorus ratio (Ca/P) of the pristine monetite synthesized phase was around the same value that is theoretically expected for monetite, i.e., ≈ 1:1 (Table 1). In addition, EDX area mapping confirmed that the elements were homogeneously distributed throughout the synthesized compounds (Figure S2). Two of the doped phases, Zn-M and Ag-M, exhibited their Ca/P values around the same values of monetite with a negligible variation. Only Sr-M showed a lower Ca/P ratio. In addition, zinc was the most incorporated element into the monetite (20 atomic %), followed by silver (7.3 atomic %), and the least incorporated element was strontium (4.5 atomic %) (Table 1). On the other hand, there was an almost complete absence of calcium in Ag-P and Zn-P, which was not the case for Sr-P with 13 atomic % of calcium, whereas Ag, Zn, and Sr were 74, 65, and 33 atomic % in these samples, respectively (Table S1).

**Table 1:** Normalized Average Weight and Atomic Percentages of the Elements: Phosphorus, Calcium, Silver, Strontium, and Zinc from Monetite and the Three Doped Phases Based on EDX Elemental Analysis^a^

composite	element	weight %	st. dev.	atomic %	st. dev.
monetite	P K	43.82	0.55	50.23	0.34
	Ca K	56.18	0.25	49.77	0.48
Ag-monetite	P K	35.94	1.77	47.18	1.56
	Ca K	44.91	1.14	45.59	1.10
	Ag L	19.15	2.12	7.24	0.93
Sr-monetite	P K	44.04	2.51	53.13	2.89
	Ca K	45.72	8.41	42.38	2.28
	Sr L	10.27	7.96	4.49	0.85
Zn-monetite	P K	31.66	8.24	41.95	9.92
	Ca K	37.14	9.86	38.28	10.31
	Zn K	31.20	6.18	19.77	4.56

a L lines are used in the quantification instead of the K lines wherever elemental overlaps may occur.

**Figure 2:**
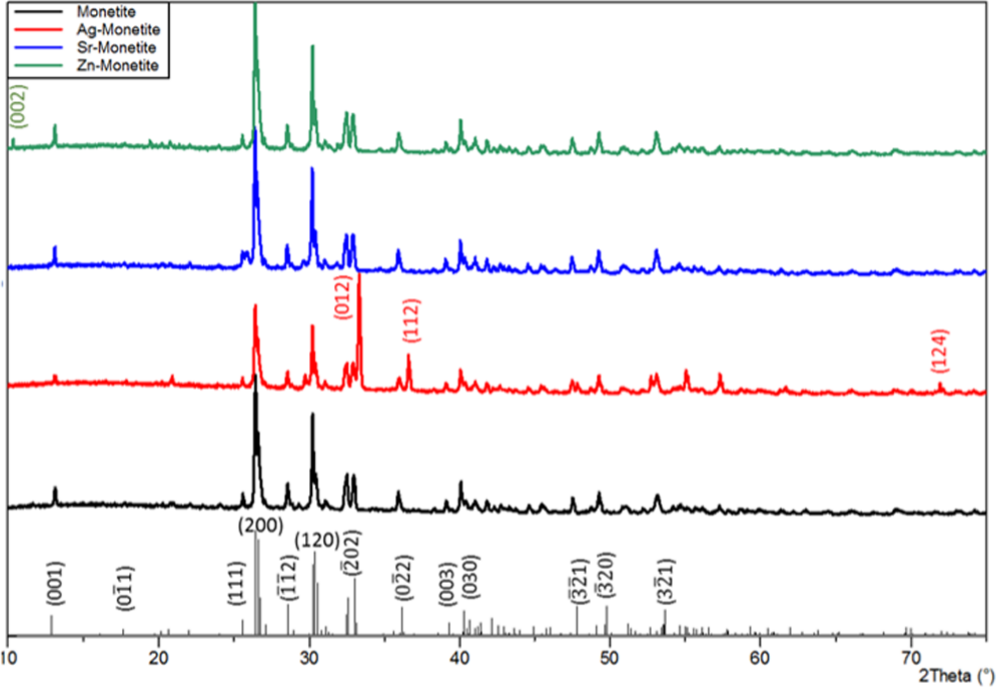
Powder X-ray diffraction (PXRD) patterns of pristine monetite (black) and its silver (red), strontium (blue), and zinc (green) doped counterparts. The theoretical PXRD pattern of monetite (lower panel, COD 9007619: triclinic, *P*1̅, *a* = 6.91 Å, *b* = 6.627 Å, *c* = 6.998 Å, α = 96.34°, β = 103.82°, γ = 88.33°). In the PXRD pattern of Ag-M (red), the peaks at 2θ 33.34°, 36.63°, and 72.02° correspond to the (012), (112) and (124) planes in Ag_3_(PO_4_) (COD 1007043: cubic, *P*4̅3*n*, *a* = 6.01 Å). The peak at 2θ 10.83° in the PXRD pattern of Zn-M corresponds to the (002) plane in Zn(PO_3_)_2_ (COD 1007095, monoclinic, C1c1, *a* = 7.66 Å, *b* = 7.61 Å, *c* = 16.34 Å, β = 92.19°).

Powder X-ray diffraction (PXRD) confirmed the successful synthesis of monetite (Figure 2) since the obtained PXRD pattern (black) coincided perfectly with that in the crystallographic database (COD ID: 9007619). The PXRD patterns of the doped phases: Ag-M (red), Sr-M (blue), and Zn-M (green) highly resembled that of the synthesized monetite, especially Sr-M that did not show any variation. However, the PXRD pattern of Ag-M showed the appearance of three additional peaks at 2θ values of 33.34, 36.63, and 72.02°, which correspond to the reflections (012), (112), and (124) in the cubic crystalline structure of Ag_3_(PO_4_) (COD ID: 1007043). In addition, the PXRD pattern of Zn-M has an additional peak at a 2θ of 10.83°, which corresponds to the reflection (002) in the monoclinic crystalline structure of zinc catena-phosphate with the chemical formula Zn(PO_3_)_2_ (COD ID: 1007095). On the other hand, the phases that were doped at higher molar ratios (Ag-P, Sr-P, and Zn-P) resulted in different PXRD patterns. Ag-P has a pattern that coincides perfectly with that of Ag_3_(PO_4_) (COD ID: 1007043), whereas Zn-P has a multiple-phase pattern and the Sr-P pattern highly resembles the PXRD pattern of pristine monetite (Figure S3).

**Figure 3:**
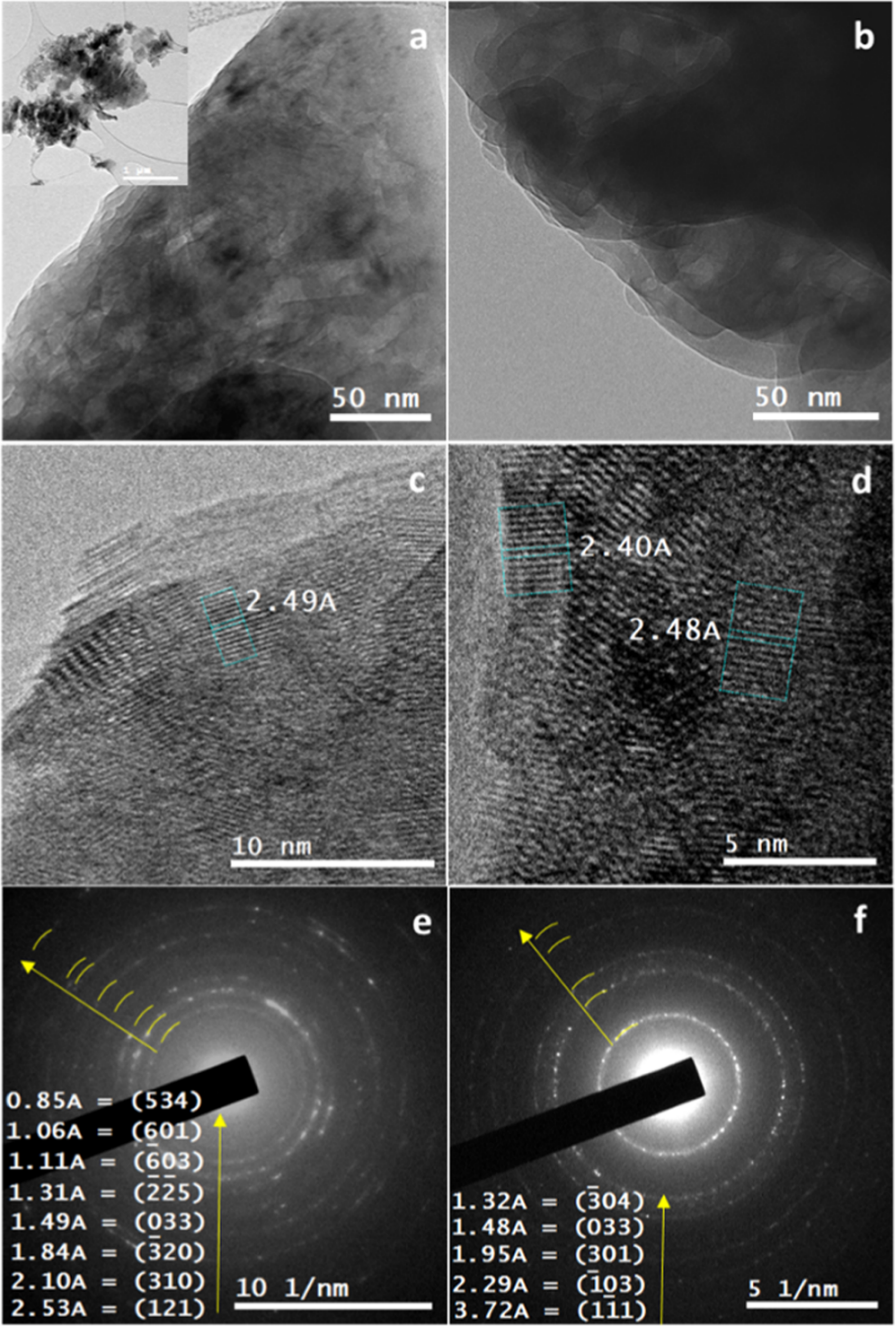
(a, b) Cryo-TEM micrographs for monetite particles showing their stacked layered platelike structures. (c, d) Cryo-HRTEM micrographs for monetite showing the lattice fringes that confirm their crystallinity with d*_hkl_* corresponding to monetite (COD 9007619). (e, f) SAED patterns for monetite revealed in the form of ring patterns with d-spacings corresponding to polycrystalline reflections of monetite (COD 9007619: triclinic, *P*1̅, *a* = 6.91Å, *b* = 6.627 Å, *c* = 6.998 Å, α = 96.34°, β = 103.82°, γ = 88.33°).

Using transmission electron microscopy (TEM), the synthesized phases were studied at the nanoscale. Due to the instant beam damage, the samples were investigated at cryogenic conditions. Cryo-TEM inspection confirmed the morphological observations determined using SEM (Figure 1) and showed a layered platelike structure of the synthesized monetite (Figure 3a,b). In addition, cryo-high-resolution TEM (cryo-HTREM) imaging showed the lattice fringes in these layered structures, confirming their crystallinity with an interlattice spacing (d_*hkl*_-spacings) corresponding to that d_*hkl*_-spacings of monetite (Figure 3c,d). Selected area electron diffraction (SAED) resulted in polycrystalline diffraction patterns that bear reflections that can be certainly attributed to monetite (COD ID: 9007619) (Figure 3e,f).

**Figure 4:**
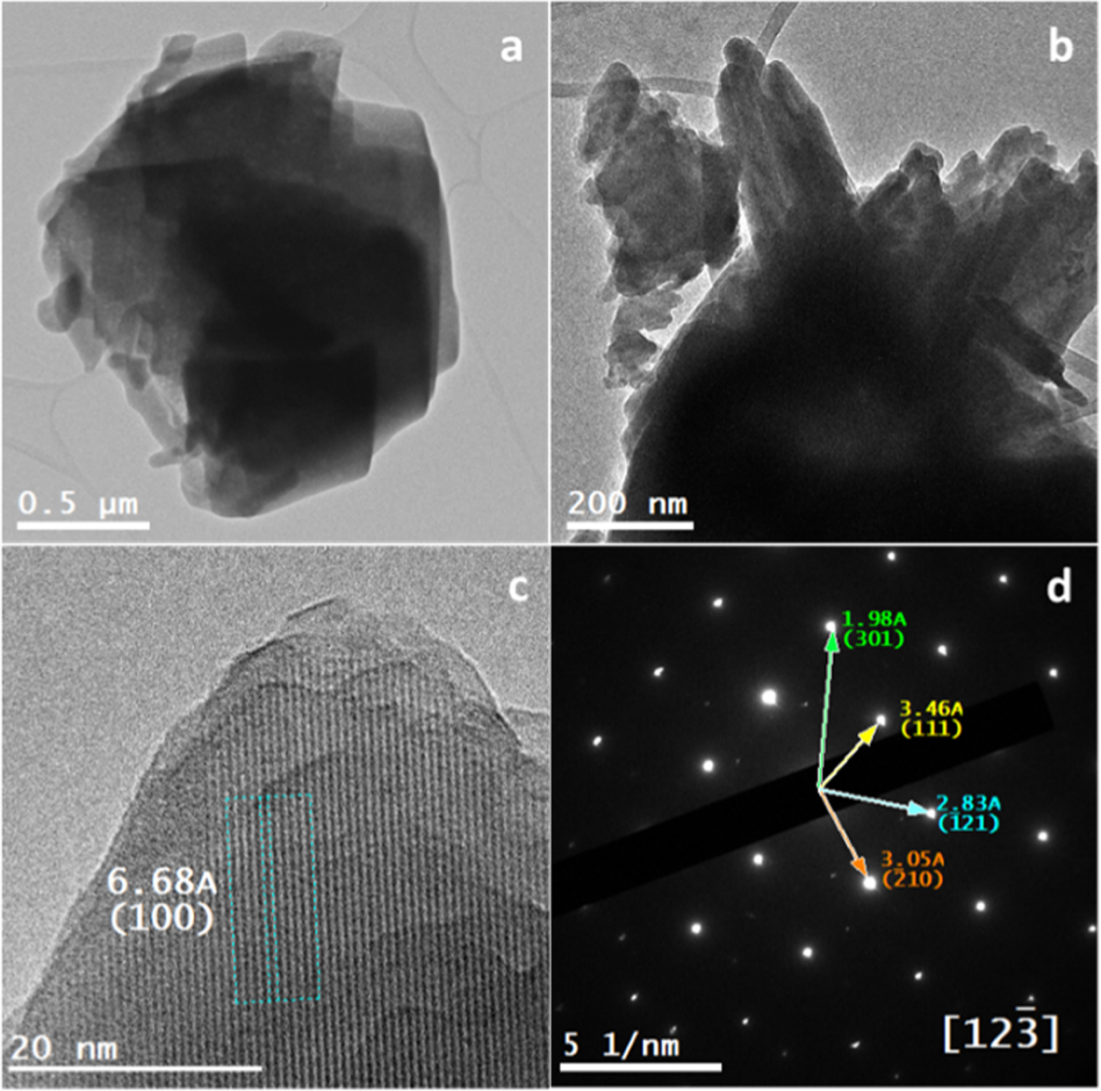
(a, b) Cryo-TEM micrographs for Sr-M showing its stacked layered platelike structures. (c) Cryo-HRTEM micrographs for Sr-M showing the lattice fringes that confirm its crystallinity with a d_*hkl*_ of 6.68 Å corresponding to the (100) plane in monetite (COD 9007619). (d) SAED pattern for Sr-M revealed in the form of a single-crystal pattern that was indexed in the zone axis [123̅] with slight expansion in the (1̅21) (2.83 Å instead of 2.71 Å) and in the (2̅10) (3.05 Å instead of 2.98 Å) in the monetite (COD 9007619: triclinic, *P̅*1, *a* = 6.91Å, *b* = 6.627 Å, *c* = 6.998 Å, α = 96.34°, β = 103.82°, γ = 88.33°).

Confirming the morphological observations realized at low magnification using an SEM (Figure 1), the cryo-HRTEM revealed the same for the Ag-M, Sr-M, and Zn-M, i.e., stacked layered platelike structures (Figure 4a,b). However, for the Sr-M and as was shown with cryo-HRTEM (Figure 4c) and SAED (Figure 4d), there was a little expansion in the d-spacing values, although the monetite structure was preserved.

### 3.2 Antimicrobial Activity and Cytocompatibility Aspects

**Figure 5:**
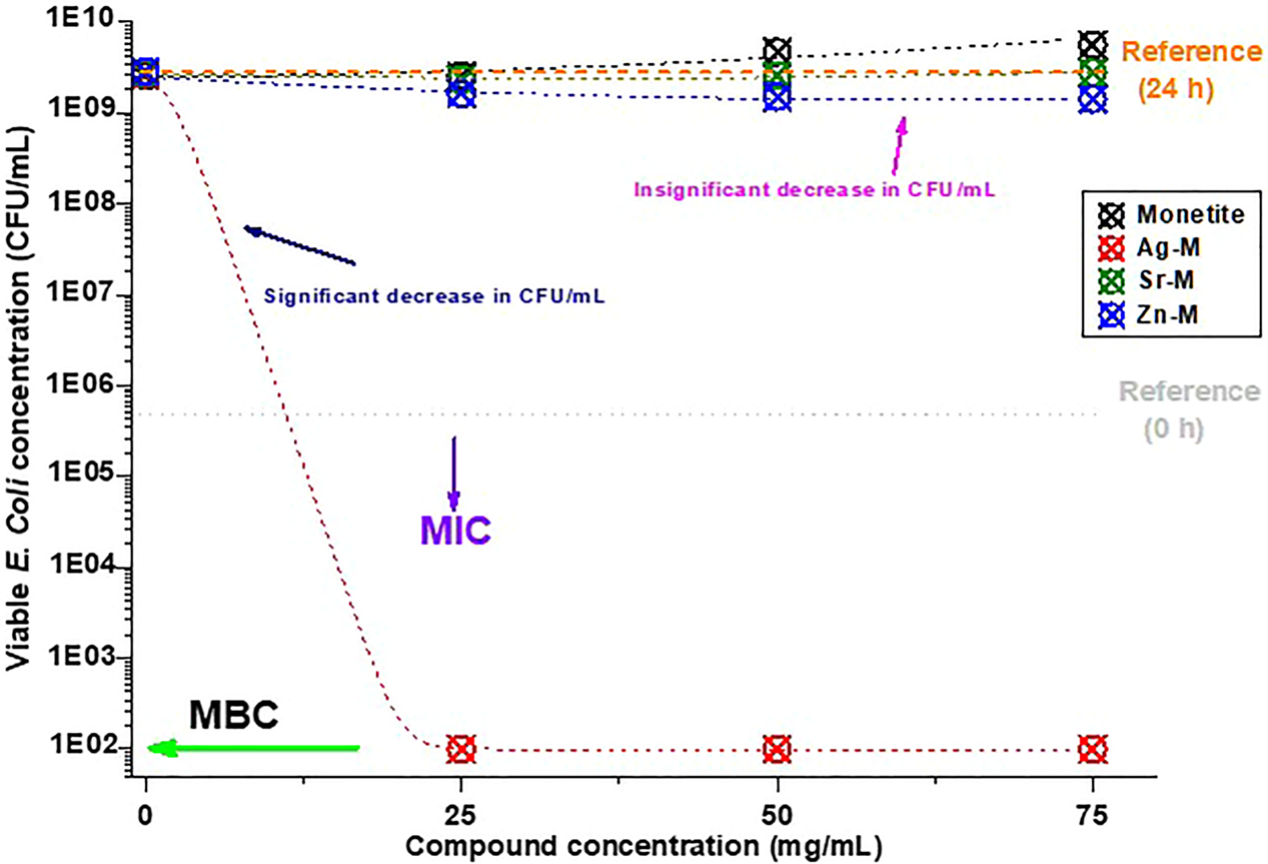
Graphical representation of the results of the assay performed to determine the antimicrobial activity of immobilized synthesized compounds: monetite (black), Ag-M (red), Sr-M (green), and Zn-M (blue); under dynamic contact conditions against *E. coli* ATCC 8739. The gray dotted line represents the initial concentration of colony-forming units (CFU/mL) before the incubation and the orange dotted line represents the CFU concentration after 24 h in the blank samples.

Monetite did not show any inhibition in bacterial growth; in contrast, its presence led to higher bacterial growth when compared to blank samples, although all samples were autoclaved beforehand (Figure 5). On the other hand, Sr-M (Sr = 4.5 atomic %) did not show any significant antimicrobial effect; only the CFU concentrations were slightly lower than those of the blank and monetite samples. In that respect, Zn-M (Zn = 19.77 atomic %) was slightly better than Sr-M in its antimicrobial activity, but the values were not of a significant effect (Figure 5). Solely Ag-M (Ag = 7.24 atomic %) effectuated a significant antimicrobial effect and led to a bactericidal effect starting from the lowest tested concentrations. Therefore, up to 75 mg/mL concentrations, only Ag-M proved its antimicrobial effectiveness (Table 2).

**Table 2:** Antimicrobial Activity of Monetite and Its Ag-, Sr-, and Zn-Doped Phases^aT^

composite	MIC	MBC	X atomic %
monetite	>75	>75	0
Ag-M	≤25	≤25	7.3
Ag-P	≤25	≤25	73.5
Sr-M	>75	>75	4.5
Sr-P	50	>75	32.9
Zn-M	>75	>75	19.8
Zn-P	25	50	65.0

aT The values indicated, in mg/mL, are the concentrations of materials that induced inhibition (MIC) and bactericidal effect (MBC) on the *E. coli* ATCC 8739. For guidance, the normalized atomic % values of X = Ag, Sr, Zn are shown.

**Figure 6:**
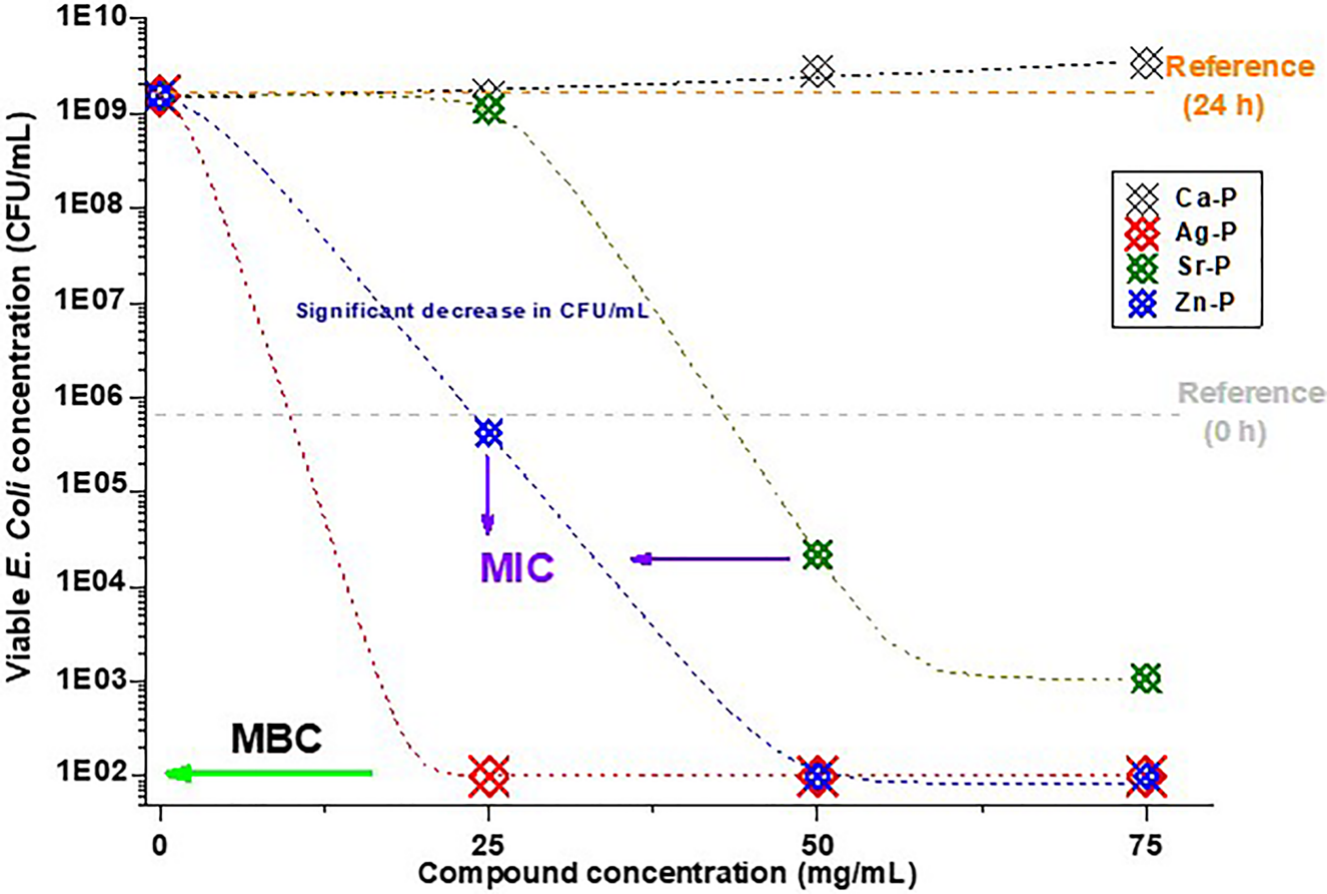
Graphical representation of the results of the assay performed to determine the antimicrobial activity of immobilized synthesized compounds: monetite (black), Ag-P (red), Sr-P (green), and Zn-P (blue); under dynamic contact conditions against *E. coli* ATCC 8739. The gray dotted line represents the initial concentration of colony-forming units (CFU/mL) before the incubation and the orange dotted line represents the CFU concentration after 24 h in the blank samples.

On the flip side, Ag-P, Sr-P, and Zn-P, in which these functional ions comprise high percentages of their content, showed much better antimicrobial activity (Figure 6). The antimicrobial effect of Ag-P (Ag = 73.5 atomic %) is the best with the bactericidal effect achieved at the lowest tested concentration in this assay (25 mg/mL). This indicates that the MIC and MBC of Ag-M and Ag-P could be induced at much lower concentrations than those used in the current study (Table 2). Zn-P (Zn = 65 atomic %) led to a significant inhibition in bacterial growth at a concentration of 25 mg/mL and a bactericidal effect at 50 mg/mL. However, Sr-P (Sr = 33 atomic %) effectuated the significant inhibition of bacterial growth starting from 50 mg/mL concentration without realizing the complete bactericidal effect up to the highest tested concentration in this assay (75 mg/mL) (Figure 6).

As per the cytocompatibility of the different phases with Saos-2, although it was proven for monetite up to a concentration of 75 mg/mL, only Zn-M showed cytocompatibility up to that concentration (Table 3). At concentrations of 50 mg/mL, apart from monetite and Zn-M that continued to show excellent cytocompatibility, Sr-M and Zn-P also exhibited acceptable levels of cytocompatibility. Up to 25 mg/mL, all zinc- and strontium-containing phases were biocompatible. On the other hand, Ag-M and Ag-P were clearly toxic to cells at concentrations higher than 5 mg/mL and 500 μg/mL, respectively.

**Table 3:**
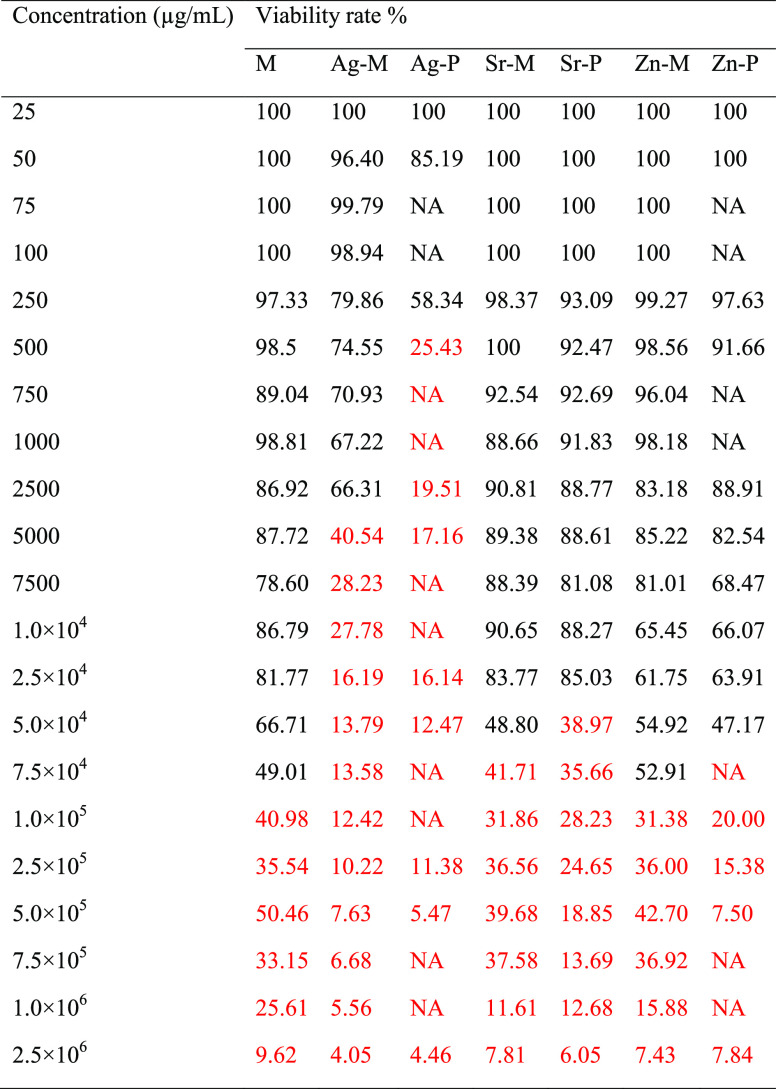
CCK-8 Proliferation Assay of Saos-2 Cultured on Different Concentrations of Samples after Incubation for 7 days^a^

a The displayed average values are for the viability rate calculated as the percentage ratio of the absorbance at wavelength 480 nm for the samples to the corresponding absorbance for control cells incubated without samples in the same microplate. The standard deviation varied between 5 and 7%. Cell death was the main regime in the values presented in red as per the color observations.

## 4 Discussion

Monetite is a resorbable CPC with useful applicability as bone cements and scaffolds and possesses more stability compared to its hydrous counterpart, brushite, from which it can be thermally prepared into different forms and sizes.^41−43,47^ An attempt was made to functionalize monetite through doping its structure with biofunctional elements known for their antimicrobial and osteoinductive properties. Although the experiments conducted to synthesize the doped phases of monetite were synthesized such that the molar ratio of Ca to Ag/Sr/Zn would be 1–0.15, the resultant phases largely varied from these values, as was revealed using EDX analysis, which allowed the precise determination of the doping efficacy at the small and large scales of the samples (Table 1). This can be attributed to their atomic radii (Table 4).^61,62^ Because of their smaller atomic radii compared to that of calcium, zinc and silver could be incorporated interstitially into the crystal structure of monetite with a probability of zinc incorporation higher than that of silver. It is worth mentioning that calcium is more reactive in single replacement reactions than zinc and silver.^63^ On the other hand, strontium could only be incorporated substitutionally into monetite because its atomic radius is larger than that of calcium and it has higher reactivity in single replacement reactions. This explanation coincides with what was concluded elsewhere.^64^ This reasoning clearly explains why strontium was the least incorporated element and zinc was the most incorporated one into the monetite. In addition, it explains the slight expansion detected in the crystalline lattice of Sr-monetite as detected in the cryo-HRTEM inspection (Figure 4).

**Table 4:** Variation of the Atomic Radii and Chemical Reactivity^63^ Given in Score (1 is the Highest and 4 is the Lowest) of the Different Elements

element	atomic number	atomic radius (Å)	chemical reactivity
calcium	20	1.97	2nd
zinc	30	1.38	3rd
silver	47	1.44	4th
strontium	38	2.15	1st

According to the combined results of the antimicrobial activity and cytocompatibility assays, zinc seems to be the most biocompatible element when compared to strontium and silver, even though the zinc/Zn-M ratio is higher than those of strontium/Sr-M or silver/Ag-M (Table 1). This, in turn, confirms the suitability of zinc as an ion/element for precautionary antimicrobial biomaterials applications. Since Zn-P effectuated a bactericidal effect at a concentration of 50 mg/mL at which the phase is still biocompatible (Tables 2 and 3), this phase could be selected as a perfect antimicrobial and biocompatible phase. In that respect, Zn-M could be considered as a biocompatible phase that could only slightly lower a possible bacterial growth.

The strontium-doped phases exhibited comparable cytocompatibility to the zinc-doped phases. However, the antimicrobial activities of the former were weaker than those of the latter. The fact that Sr-P, according to its PXRD pattern and morphological assessments, is a monetite phase with high strontium content only resulted in a significant inhibition in bacterial growth at a concentration ≥ 50 mg/mL (Figure S2 and Table S1). This indicates the effectiveness of strontium ions as an antimicrobial agent at relatively high concentrations, as in the case of Sr-P, and implies that the weak antimicrobial activity of Sr-M and Sr-P could be attributed to the low content of strontium (Sr = 4.5, 32.5 atomic %, respectively) in the resultant synthesized phase, compared with Zn-M and Zn-P (Zn = 20, 65 atomic %, respectively) (Tables 1 and S1). Yet, the presence of strontium in even very low percentages could slightly decrease the bacterial growth (Figures 5 and 6). These results confirm the antimicrobial activity of strontium in addition to its widely known osteoinductivity, which was previously reported upon their incorporation into bioglasses.^65,66^

On the other hand, silver-doped phases showed the best antimicrobial effect as expected,^67^ even though its biocompatibility is limited to low concentrations.^68^ However, the effectiveness of silver as an antimicrobial agent is widely reported to be in the microgram range of concentrations.^69^ Moreover, we found that doping calcium phosphate phases with silver led to the production of compounds with much better cytocompatibility than those produced relying on the other metal phosphates (zirconium phosphate and titanium phosphates).^49,58^ This can be attributed to the closer biological resemblance of calcium phosphates as a carrier to these ions compared to the other metal phosphates.

Finally, it should be noted here that the synthesized phases were tested in their powder forms and not in their scaffold configuration. This could have significantly decreased the acquired viability rates for the respective phases because of the higher abundance of phosphorus ions that are known to significantly increase the rate of cell apoptosis.^70^ However, this should not be the actual effect if monetite scaffolds are designed as implants with the consequent more controlled release of phosphorus ions.

## 5 Conclusions

In this study, we report on the synthesis and biofunctionalization of an important resorbable phase of calcium phosphate known as monetite (anhydrous dicalcium phosphate), which is more stable and practical in designing biomaterials implants when compared to the widely studied brushite (hydrous dicalcium phosphate). Using different proportions of silver, strontium, and zinc nitrate, six different doped phases were synthesized (Ag-M, Sr-M, Zn-M, Ag-P, Sr-P, and Zn-P). A thorough structural study revealed the nature and actual incorporation of the biofunctional elements at the nanoscale. The biocompatibility and antimicrobial activity investigations confirmed the effectiveness of silver ions containing composites as outstanding antimicrobial composites limited to low concentrations (≤5 mg/mL). Strontium, which has been widely accepted as an osteoinductivity-promoting element, has been proven here to increase the antimicrobial effectiveness of monetite but at high concentrations (Sr = 33 atomic %). In between comes zinc, as a better antimicrobial element than strontium and a better biocompatible element than silver. The structural investigations revealed the facile incorporation of zinc into the monetite crystalline lattice without compromising its structure. Furthermore, its presence in concentrations of 20 and up to 65 atomic % in the resultant phases, through increasing the molar ratio of zinc nitrate to monetite during the doping process, achieved significant inhibition of bacterial growth without inducing cytotoxicity up to reasonable practical concentrations. Therefore, this research confirms the merits of functionalizing monetite and the antimicrobial effectiveness of silver incorporation, but it also highlights, in particular, the potential beneficial combination of metal phosphates with zinc and strontium.

## Supporting Information Available

SEM micrographs of Ag-P, Sr-P, and Zn-P (Figure S1); EDX area mapping showing the homogeneous distribution of Ag, Sr, and Zn in phosphate phases (Figure S2); powder X-ray diffraction (PXRD) patterns of Ag-P, Sr-P, and Zn-P (Figure S3); and normalized average weight and atomic percentages of phosphorus, calcium, silver, strontium, and zinc from the three highly doped phases based on EDX elemental analysis (Table S1) (PDF)

## Notes

Dr. Adawy previous email address: hassanalaa@uniovi.es
