# Supplementary material for: Probing the Structure, Cytocompatibility, and Antimicrobial Efficacy of Silver-, Strontium-, and Zinc-Doped Monetite
Source: ACS Appl Bio Mater. 2022 Mar 24;5(4):1648–57. doi: 10.1021/acsabm.2c00047 (PMC9019811; doi:10.1021/acsabm.2c00047)
Supplement: Supplementary file 1 — mt2c00047_si_001.pdf [file mt2c00047_si_001.pdf]

Supporting Information for

Probing the Structure, Cytocompatibility and Antimicrobial Efficacy of Silver-, Strontium-, and Zinc-doped Monetite

Alaa Adawy[1]*, Raquel Diaz[2]

[1] Unit of Electron Microscopy and Nanotechnology, Institute for Scientific and Technological Resources (SCTs), University of Oviedo, 33006, Oviedo, Asturias, Spain
[2] Nanomaterials and Nanotechnology Research Centre—CINN (CSIC), 33940 El Entrego, Spain
*Correspondence: UO263127@uniovi.es; a.adawy@outlook.com; Tel.: +34 9 8510 5456

Supplementary figures

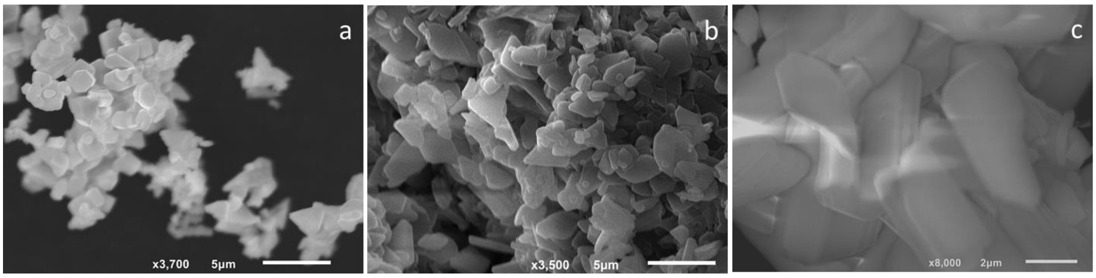

Figure S1. SEM micrographs the Ag-P (a); Sr-P (b); and Zn-P (c)

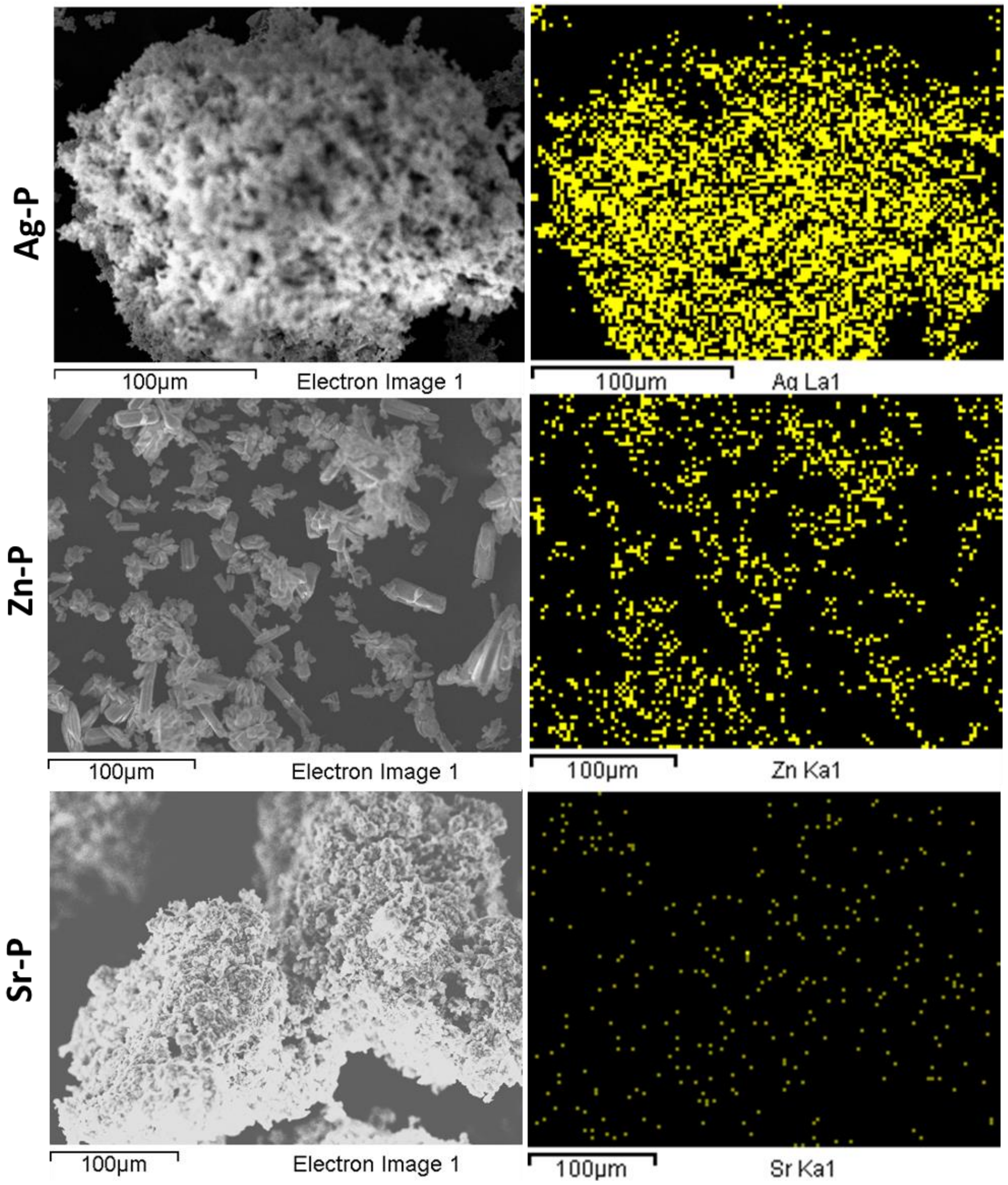

Figure S2. EDX area mapping of the doped phases at 1:1 molar ratio. As revealed with the quantitative analysis, the most incorporated element was silver (75 % atomic), followed by zinc (65 % atomic), and the least was strontium (33 %atomic). The maps confirm at the large scale the homogenous distribution of these elements in the resultant compounds.

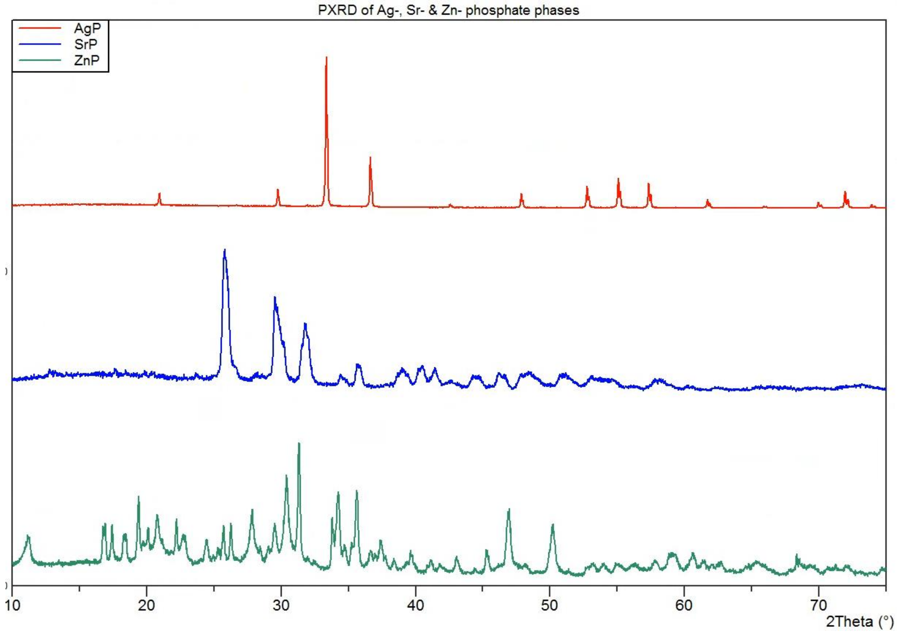

Figure S3. Powder X-ray diffraction (PXRD) patterns of Ag-P (red), Sr-P (blue), and Zn-P (green). PXRD pattern of Ag-P coincided perfectly with the theoretical PXRD pattern of $Ag_3(PO_4)$ (COD 1007043: Cubic, P$\overline{4}$3n, a = 6.01Å. For Zn-P, PXRD pattern is a mixture of monetite (COD 9007619: Triclinic, P$\overline{1}$, a = 6.91Å, b = 6.627 Å, c = 6.998 Å, α = 96.34°, β = 103.82°, γ = 88.33°) and $Zn(PO_3)_2$ (COD 1007095, Monoclinic, C1c1, a = 7.66 Å, b = 7.61 Å, c = 16.34 Å, β = 92.19°). PXRD pattern of Sr-P is closer to pure monetite, although strontium is greatly incorporated.

Table S1: Normalized average weight and atomic percentages of the elements: phosphorus, calcium, silver, strontium, and zinc from the three highly doped phases based on EDX elemental analysis

**Composite**	**Element**	**Weight %**	**Atomic%**
**Ag-phosphate**	P K	9.03	25.51
	Ca K	0.47	1.02
	Ag L	90.51	73.46
**Sr-phosphate**	P K	32.84	53.89
	Ca K	10.40	13.118
	Sr L	56.76	32.93
**Zn-phosphate**	P K	20.19	34.76
	Ca K	0.25	0.33
	Zn K	79.56	64.91
